# Optimization of Astaxanthin Recovery in the Downstream Process of *Haematococcus pluvialis*

**DOI:** 10.3390/foods11091352

**Published:** 2022-05-06

**Authors:** Inga K. Koopmann, Simone Möller, Clemens Elle, Stefan Hindersin, Annemarie Kramer, Antje Labes

**Affiliations:** 1 ZAiT, Bio and Food Technology, Faculty Energy and Biotechnology, Flensburg University of Applied Sciences, 24943 Flensburg, Germany; inga.koopmann@hs-flensburg.de (I.K.K.); simone.moeller@lactotec.de (S.M.); annemarie.kramer@hs-flensburg.de (A.K.); 2 Sea & Sun Technology GmbH, 24610 Trappenkamp, Germany; elle@sea-sun-tech.com (C.E.); hindersin@sea-sun-tech.com (S.H.)

**Keywords:** isomerization, UHPLC-PDA-MS, microalgae, carotenoids, disruption, drying, supercritical CO_2_ extraction, economic feasibility

## Abstract

Astaxanthin derived from *Haematococcus pluvialis* is a valuable metabolite applied in a wide range of products. Its extraction depends on a sophisticated series of downstream process steps, including harvesting, disruption, drying, and extraction, of which some are dependent on each other. To determine the processes that yield maximum astaxanthin recovery, bead milling, high-pressure homogenization, and no disruption of *H. pluvialis* biomass were coupled with spray-drying, vacuum-drying, and freeze-drying in all possible combinations. Eventually, astaxanthin was extracted using supercritical CO_2_. Optimal conditions for spray-drying were evaluated through the design of experiments and standard least squares regression (feed rate: 5.8 mL/min, spray gas flow: 400 NL/h, inlet temperature: 180 °C). Maximal astaxanthin recoveries were yielded using high-pressure homogenization and lyophilization (85.4%). All combinations of milling or high-pressure homogenization and lyophilization or spray-drying resulted in similar recoveries. Bead milling and spray-drying repeated with a larger spray-dryer resulted in similar astaxanthin recoveries compared with the laboratory scale. Smaller astaxanthin recoveries after the extraction of vacuum-dried biomass were mainly attributed to textural changes. Evaluation of these results in an economic context led to a recommendation for bead milling and spray-drying prior to supercritical CO_2_ extraction to achieve the maximum astaxanthin recoveries.

## 1. Introduction

Astaxanthin (3,3′-dihydroxy-β,β′-carotene-4,4′-dione), a secondary ketocarotenoid, can reduce (photo-)oxidative stress by scavenging a variety of radicals and quenching singlet oxygen [1,2,3,4,5,6]. This strong antioxidant capability applies in vivo and in vitro [7,8,9,10,11,12]. Astaxanthin has been used as a nutritional supplement, food and feed additive, and in cosmetics. Beneficial health effects, such as cardiovascular disease prevention and improved immune response, have been associated with astaxanthin consumption. The approval of astaxanthin from natural sources for human consumption has been granted in many countries worldwide, with recommended daily intakes of up to 24 mg/day permitted by the United States Food and Drug Administration [13]. Although it can be produced synthetically, its natural form has gained interest with respect to consumer demands [14,15]. Various organisms have the potential to accumulate astaxanthin. Its biosynthesis has mainly been observed in microalgae [16,17,18,19,20,21,22,23,24], but also in a few protists [25,26,27], bacteria [28,29,30,31,32], archaea [33], as well as yeasts [34,35], and very few plant species [36,37]. A major source for the biotechnological production of astaxanthin is the green alga *Haematococcus pluvialis* [24,38]. It forms flagellated, motile cells under optimal environmental conditions. These transform into spherical, non-motile aplanospores, which accumulate astaxanthin under stress conditions such as high light irradiation or nutrient deficiency [24,39,40,41,42,43]. *H. pluvialis* can accumulate 1.9% to 7.0% astaxanthin of its dry weight [44,45,46,47,48,49].

Astaxanthin often has to be extracted from whole-cell biomass to meet legal and processing demands, e.g., raw materials free from cellular debris are needed for cosmetics or feed additive production. Therefore, elaborate downstream processing is necessary, which may constitute a large part of the total production costs [50,51,52,53]. Depending on the purity desired, it generally comprises four or more steps: harvesting the biomass, cell disruption, drying, and the extraction of astaxanthin [50]. Harvesting comprises biomass concentration to reduce the volume in further processes.

Disruption is necessary to facilitate later accessibility during the extraction [54,55], because the aplanospores of *H. pluvialis* have a very thick and rigid cell wall, limiting extractability [56,57,58]. The disrupted biomass is dried to enhance its shelf life and prepare for extraction, which is the last step. Various methods have been described to achieve these different goals, and some even combine them. Their applicability on an industrial scale is often limited. E.g., chemical extraction approaches using methanol, ethanol, acetone, acetonitrile, hexane or hydrochloric acid may be suitable on a laboratory scale [58,59,60,61], but are restricted in human applications due to possible health risks, product degradation [61,62], and environmental issues. Physical and biological treatments using sonication, microwaves, autoclaving, ionic liquids, enzymatic treatment or germination [59,62,63,64,65,66] often lack efficiency, are difficult to upscale, or are too expensive. Simple mechanical disruption processes such as milling [38,53] or high-pressure homogenization (HPH) [67,68] are possible, but risk astaxanthin degradation. Additionally, HPH is quite expensive.

Combined disruption and extraction have been described with generally recognized as safe (GRAS) solvents [69,70], but the selectivity of these extraction processes is limited, compared with, e.g., supercritical CO_2_ (SC-CO_2_) extraction. Drying techniques such as freeze-drying (FD), spray-drying (SD), and belt-drying have been applied for further dehydration of the biomass [53,71,72,73]. In all these processes, attention must be paid to product degradation [74], because astaxanthin is prone to oxidation [75]. This is especially important when working at higher temperatures. Final astaxanthin extraction can be achieved with various solvents. Reductions in the use of fossil-based solvents is an important environmental goal. It must also be considered that consumers demand increasing standards of sustainability, eco-friendliness and product safety. SC-CO_2_ extraction enables the gentle, selective, and efficient recovery of astaxanthin from other, polar compounds and cell biomass while being non-toxic, aseptic, and environmentally friendly. Accordingly, many authors have proposed SC-CO_2_ extraction for safe and environmentally friendly production of astaxanthin-containing oleoresins [54,55,76,77,78,79,80,81,82,83,84,85,86].

Of the named processes, bead milling (BM), SD, and SC-CO_2_ extraction are commonly applied and discussed for the industrial and pilot-scale production of astaxanthin, as well as for economic assessments [50,52,53,73,87]. Many of those processes have been investigated individually concerning their efficiency and, most importantly, their astaxanthin recovery. Nevertheless, only a few studies have combined the various processes used in downstream processing. Therefore, this study combined different disruption and drying methods for *H. pluvialis* biomass in all possible combinations.

## 2. Materials and Methods

### 2.1. Chemicals and Reagents

Analytical-grade acetone, petroleum ether and hypergrade acetonitrile were obtained from Merck (Darmstadt, Germany). Ethanol and Tris(hydroxymethyl) aminomethane (TRIS) (≥99.9%) were provided by Carl Roth (Karlsruhe, Germany), and formic acid (99% ULC/MS) by Biosolve (Valkenswaard, The Netherlands). Cholesterol esterase from *Pseudomonas* sp. was purchased from MP Biomedicals (Eschwege, Germany). All-*E*-astaxanthin standard in its free form (SML0982, ≥97%, 3S,3′S, from *Blakslea trispora*) was obtained from Sigma-Aldrich (Taufkirchen, Germany), and astaxanthin monopalmitate (1017, 3RS, 3′RS) was provided by CaroteNature (Münsingen, Switzerland).

### 2.2. Haematococcus Pluvialis Cultivation

*Haematococcus pluvialis* (proprietary strain of Sea & Sun Technology, Trappenkamp, Germany) was cultivated indoors in a glass tube system of 1500 L under artificial light conditions (LED tubes, 24 h) at 23 ± 1 °C. The cells were cultivated in BG11 medium consisting of 17.6 mM NaNO_3_, 0.18 mM K_2_HPO_4_ · 3 H_2_O, 0.3 mM MgSO_4_ · 7 H_2_O, 0.25 mM CaCl_2_ · 2 H_2_O, 0.031 mM citric acid, 0.023 mM ferric ammonium citrate, 0.003 mM Na_2_EDTA · 2H_2_O, 0.19 mM NaCO_3_ and 1 mL/L trace metal solution made of 1.0 mM H_3_BO_3_, 1.0 mM MnSO_4_ · H_2_O, 1.00 mM ZnSO_4_ · 7 H_2_O, 0.01 mM (NH_4_)_6_Mo_7_O_24_ · 4 H_2_O and 0.1 mM CuSO_4_ · 5 H_2_O until nitrogen was completely depleted. Subsequently, the cells were transferred to a 3000 L glass tube system, operated in a greenhouse (Gönnebek, Germany), and exposed to direct sunlight to induce astaxanthin production. The cells were cultivated in the greenhouse until the astaxanthin content reached its highest level. The cells were harvested by centrifugation (Clara 80, Alfa Laval, Lund, Sweden). The harvested biomass had a final concentration of 179.9 ± 0.05 g/L (*n* = 3).

### 2.3. Downstream Processing—Laboratory Scale

#### 2.3.1. Disruption of *H. pluvialis* Biomass

Two disruption methods, i.e., bead milling (BM) and high-pressure homogenization (HPH), were compared.

An agitator bead mill (Dyno-Mill KDL A, Willy A. Bachofen AG, Muttenz, Switzerland) was used for cell disruption by BM. The grinding chamber had a volume of 600 mL and was filled to 85% with grinding beads (0.8–1.0 mm diameter, 83% ZrO_2_ and 17% CeO_2_). It was cooled by cold water flowing through a double jacket, resulting in a biomass temperature between 36 °C and 44 °C in the outlet. The separation gap width was 0.2 mm. The peripheral speed for the agitator discs (64 mm diameter) was 14 m/s. The throughput was between 10.5 L/h and 12.0 L/h, resulting in average dwell times between 1.4 and 1.7 min for one passage. All batches were milled three times, and samples were taken after each passage. The aliquots were homogenized after the experiment and stored at −21 °C, prior to astaxanthin analysis and further processing.

For HPH, the biomass with a concentration of 180 g/L was diluted 1 to 4 with water and filtered. This was the highest concentration homogenizable with this device. The biomass was filtered through 50 µm polyamide gauze. Four aliquots of 500 to 600 mL were homogenized twice separately at approximately 80 MPa (EmulsiFlex-C3, Avestin, Mannheim, Germany). Samples were taken and measured before and after each disruption step. Cell disintegration and dry weight were determined directly after the experiments. The aliquots were homogenized after the experiment and stored at −21 °C, prior to astaxanthin analysis or further processing.

#### 2.3.2. Drying of *H. pluvialis* Biomass

Three drying techniques were compared: spray-drying (SD), freeze-drying (FD), and vacuum-drying (VD).

SD conditions were assessed by the design of experiments for optimal experimental parameters to dry 200 mL continuously agitated, non-disrupted *H. pluvialis* biomass (concentration 99.4 g/L) at the laboratory scale with a mini spray-dryer B-191 (Büchi, Essen, Germany) for maximization of the biomass and astaxanthin yield. Therefore, the amount of dried biomass in the collection container and its astaxanthin content were measured and used independently as dependent variables. Three independent variables were chosen and tested in ten different scenarios at distinct points: spray gas flow was applied at 400 and 500 NL/h, product flow rate at 5%, 10%, and 15% ≙ 2.7, 5.8, and 8.7 mL/min, respectively, and inlet temperature at 160 and 180 °C. In three further runs, the temperature was reduced to 140 and 120 °C, and spray gas flow was increased to 600 NL/h in one run. The experiment using the parameters that were later considered optimal was repeated, resulting in 14 experiments. An overview of the different process conditions can be found in Table S1. Hot air volume flow was maintained constant at 25 m^3^/h throughout all experiments.

The obtained results were analyzed using JMP PRO software version 15.0.0. Data were regressed using standard least squares with different models, namely, linear ((Equation (1)) and quadratic (Equation (2)), as well as quadratic combined with interaction terms (response surface model) (Equation (3)). *Y* is the yield, *a* is the intercept, *b_i_, c_i_,* and *d_ij_* are model coefficients, and *X_i_* and *X_j_* represent the model regressors.

(1) $$Y\;=\;a\;+\;\overset{3}{\underset{i\;=\;1}{\sum }}b_{i}X_{i}$$

(2) $$Y\;=\;a\;+\;\overset{3}{\underset{i\;=\;1}{\sum }}b_{i}X_{i}\;+\;\overset{3}{\underset{i\;=\;1}{\sum }}c_{i}X_{i}^{2}$$

(3) $$Y\;=\;a\;+\;\overset{3}{\underset{i\;=\;1}{\sum }}b_{i}X_{i}\;+\;\overset{3}{\underset{i\;=\;1}{\sum }}c_{i}X_{i}^{2}\;+\;\overset{2}{\underset{i\;=\;1}{\sum }}\overset{3}{\underset{j\;=\;i\;+\;1}{\sum }}d_{ij}X_{i}X_{j}$$

The adjusted coefficients of determination were used to compare the three different models. The model with the highest adjusted coefficient of determination was then optimized numerically via a gradient descent algorithm on the yield. T-tests were applied to identify the statistical significance of the model parameters. The described approaches were carried out, regressing the biomass and astaxanthin contents. The results of both estimations were combined with approximate optimal parameters for astaxanthin recovery with regard to degradation and biomass yield. These parameters were verified by applying them to the drying process of the same biomass slurry in the same concentration and diluted to 50 g/L. Another *H. pluvialis* batch with a concentration of 200 g/L was used as a reference. These concentrations reflected those used in the following SD experiments. The spray nozzle temperature was regulated with an external circulation thermostat at 40 °C.

Using the optimal parameters obtained from the design of experiments, SD in the final experiment was performed using a mini spray-dryer B-191 (Büchi, Essen, Germany). Conditions were set to 180 °C inlet temperature, product flow of 5.8 mL/min, spray gas flow of 400 NL/h, and ventilation of 25 m^3^/h. The spray nozzle was tempered with an external circulation thermostat at 40 °C. Three aliquots of 200 mL of non-disrupted, milled, and high-pressure homogenized samples were used (179.9, 176.0, and 45.5 g/L, respectively). Biomass recoveries were determined in the different compartments of the spray-dryer, namely, the spray tower, the joint, the cyclone, and the collection vessel by weighing them before and after drying. Astaxanthin and dry mass content were determined in the *H. pluvialis* powder recovered from the collection vessel. This powder was also used for SC-CO_2_ extraction.

FD was performed with multiple aliquots taken from each differently disrupted batch. Three aliquots of milled biomass were used with approximately 7.04 g biomass each, and six aliquots of the samples that were disintegrated by HPH, equaling approximately 1.82 g biomass each, were applied. For comparison, three 40 mL aliquots of concentrated and non-disrupted biomass, equaling approximately 7.20 g, were used. All were poured into aluminum bowls for FD. They had a filling height of approximately 0.8 cm and were frozen at −80 °C prior to FD (Alpha 1–4, Christ, Osterode, Germany) for 12 h at 37 Pa.

VD was performed in a vacuum chamber (Vacutherm, Heraeus instruments, Hanau, Germany). Sample preparation was accomplished in the same way as for FD. In the first step, the temperature in the vacuum chamber was set to 40 °C and approximately 5–15 kPa (main drying). In the second step, the temperature was increased to 50 °C, and the vacuum was reduced to approximately 3–8 kPa (secondary drying). The samples were dried until reaching constant weight. The six samples with the higher cell concentration were dried together over 24 h with 1:1 main and secondary drying. The high-pressure homogenized samples were dried in duplets due to their higher water content over 12 h with 1:1 main and secondary drying.

#### 2.3.3. Supercritical CO_2_ Extraction of Astaxanthin

Subsequently, 125 ± 2 mg dried *H. pluvialis* powder of the previous experiments were homogenized with approximately 1.1 g ± 0.2 g glass beads and filled in a 5 mL extraction vessel. A plain layer of beads was placed on the exit of the cartridge. On top of that layer, more beads and the weighed biomass were placed alternately and gently stirred. SC-CO_2_ extraction was performed with an MV-10 ASFE system (Waters, Milford, MA, USA) at 35 MPa and 6.0 mL/min CO_2_ flow for 30 min with 1.5 mL/min ethanol as a co-solvent and an additional 0.5 mL/min ethanol as make-up solvent after depressurization at 50 °C.

Afterwards, drying and depressurization of the sample were performed in three steps, each taking two minutes: First, the co- and make-up solvent flow were reduced to zero and the pressure to 20 MPa. Second, CO_2_ flow was reduced to 5 mL/min, and the pressure was reduced to 15 MPa. Third, CO_2_ flow was reduced to 2 mL/min, and pressure was reduced to 10 MPa. Extract volume was determined by weighing, assuming a density of 0.79 kg/L, and verified by volumetric measurements. Aliquots were transferred for astaxanthin quantification. The residual extract was dried in a rotary evaporator at 40 °C and 17.8 kPa. The extract was resuspended in acetone and transferred into a weighed amber vial. Acetone was evaporated at 40 °C under a gentle stream of nitrogen, and the weight of dried extract was determined. Recoveries of astaxanthin in SC-CO_2_ extracts were calculated by comparing them to the astaxanthin content of the applied biomass before extraction.

### 2.4. Downstream Processing—Pilot Scale

Another batch of biomass was used exclusively in these experiments. This was disrupted similarly as described for the laboratory scale, using the same agitator bead mill with the same specifications as used before. The biomass was concentrated to a density of 15–18% *w*/*v*. The biomass throughput was set to 10 L/h, and three passages of the whole biomass were performed. The disrupted biomass was recovered in a light-protected stainless-steel vessel. The milled biomass was dried in a spray-dryer (Anhydro MS150, SPX FLOW, Charlotte, NC, USA) at 180 °C and a feed flow rate of 5 L/h. The biomass slurry was stirred to prevent agglomerations and clogging of the spray nozzle. The nozzle pressure was set to 0.3 MPa, and the outlet temperature was set to 90 °C. The resulting powder was collected in a stainless-steel pot at the end of the cyclone. The powder was vacuum-sealed and stored at −21 °C in the dark.

### 2.5. Disintegration Rate

The cell disintegration rate was determined visually by microscopic methods. Intact cells were counted at least in triplets before and after disruption in a Neubauer improved (BM samples) and a Fuchs–Rosenthal (HPH samples) cell counting chamber and were related to each other.

### 2.6. Dry Weight

Dry weight of the wet samples was determined after washing the samples with distilled water and drying them in a moisture analyzer MA 50/1.X2.A (Radwag, Hilden, Germany) at 140 °C until reaching constant weight (method adapted as per the manufacturer’s recommendation). Dry weight after FD and VD was determined by measuring the respective sample weight and calculating the dry weight with the help of the dry weight content of the wet biomass. Dry weight after SD was measured with 0.5 to 1.0 g sample in a moisture analyzer (MA 50/1.X2.A, Radwag, Hilden, Germany) at 140 °C until reaching constant weight. Residual moisture of all dried samples was smaller than 9%, and thus considered negligible.

### 2.7. Astaxanthin Analysis

Astaxanthin analysis was performed after each process step, according to Koopmann et al. [88], with adaptions to the differently processed biomasses as follows: wet biomass with known concentrations was weighed into lysis tubes type C (Analytik Jena, Jena, Germany) with less than 300 µL and final amounts of 0.3–2.0 mg *H. pluvialis* dry mass. Dry biomass was adjusted when dried by SD or FD. Biomass dehydrated by VD was treated equally, but it was weighed into type G bead tubes (Macherey Nagel, Düren, Germany). All samples were filled up to 500 µL with acetone, and cells were disrupted for 3 min in a swing mill (MM 2000, Retsch, Haan, Germany) at 27 Hz. Subsequently, centrifugation at 10,000× *g* and separation of carotenoid containing acetone from the residual biomass were performed. Extraction with 500 µL fresh acetone, disruption, and separation were repeated twice until the residual biomass and the acetone were colorless.

For de-esterification, the combined supernatant was filled up to 3 mL with acetone. Ethanolic SC-CO_2_ extracts were directly added to the reaction mixture in volumes of 100 to 1000 µL and filled up with acetone to 3 mL. Then, 2 mL of 50 mM TRIS buffer (pH 7 at 21 °C) and 600 µL cholesterol esterase solution with a concentration of 3.3 U/mL suspended in the same buffer were added. The tubes were incubated at 37 °C in a water bath and mixed gently every 10 min. Astaxanthin was recovered by liquid–liquid extraction with 2 mL of petroleum ether. The mixture was shaken vigorously for 10 s to ameliorate astaxanthin transfer into the petroleum ether. Subsequent phase separation was enhanced by centrifugation at 3,000× *g* for 1 min. The upper, astaxanthin-containing layer was filtered through a 0.45 µm PTFE filter, and either measured directly or stored for one night at −21 °C prior to analysis.

Extracts were vortexed and ultrasonicated for 20–30 s before analysis if stored overnight previously. Qualification and quantification of astaxanthin were performed by ultra-high-performance liquid chromatography (UHPLC). This was performed on an ACQUITY Arc system (Waters, Milford, MA, USA) coupled with a UV/Vis detector (2998 PDA Detector, Waters, Milford, MA, USA) and a mass spectrometer (Acquity QDa Detector, Waters, Milford, MA, USA), using a C18-column (Cortecs C18 2.7 µm, 90 Å, 3.0 × 100 mm, Waters, Milford, MA, USA) operated at 40 °C. The injection volume was 5 µL. A gradient of H_2_O (A) and acetonitrile (B) was applied (0 min 70% A, 4 min 10% A, 9 min 0% A, 11.5 min 70% A until 15 min) with 0.1% formic acid added to both solvents. Flow velocity was 0.5 mL/min. Optical spectra were measured in a range of 200 to 800 nm, and astaxanthin data were analyzed and quantified at 474 nm. The mass spectrometer with electrospray ionization was operated in positive mode with a cone voltage of 15 V and a probe temperature of 600 °C, measuring in a range of 150 to 1250 *m*/*z*. For further accuracy, the mass of astaxanthin was observed by selected ion recording at 598 *m*/*z* [M + H]^+^. The volume dilatation of the petroleum ether phase after liquid–liquid extraction was considered to quantify astaxanthin, de-esterified in the presence or absence of ethanol.

For identification and quantification, 0.5 to 54.8 µg free all-*E-*astaxanthin, and 1.0 to 62.6 µg astaxanthin monopalmitate were processed like in de-esterification, and astaxanthin was quantified with UHPLC-PDA. However, in the case of the free all-*E-*astaxanthin standard, the cholesterol esterase solution was replaced with the same amount of TRIS buffer. A proportion of 69.4% *w*/*w* astaxanthin was assumed for the quantification of ester-derived, free astaxanthin. Peaks with a corresponding UV/Vis absorption spectrum [89,90,91,92,93] accompanied by peaks with the mass of astaxanthin in SIR were assigned to the *Z*-isomers 9*Z-* and 13*Z*-astaxanthin and several di-*Z-*isomers. The latter are difficult to differentiate without further validation; therefore, they were summed and consecutively termed di-*Z-*isomers. Quantities of all diastereomers were estimated using the quantification of all-*E*-astaxanthin, corrected by factors adjusting the different extinction coefficients published by Bjerkeng et al. [94], namely, 1.20 for 9*Z*-astaxanthin, 1.56 for 13*Z-*astaxanthin, and 1.11 for the di-*Z-*isomers.

Linear regression of the calibration data was performed by the ordinary least squares method, and the significance of the deviation of the y-intercepts from zero was evaluated by *t*-tests [88]. Tests on significant deviations were calculated using mean difference tests with a level of significance of σ = 0.05.

### 2.8. Evaluation of Significance

All disruption processes and SD were performed at least in triplicates using identical biomass batches. FD and VD were performed once to multiple times with triplets to sextets of the samples in separated containers. SC-CO_2_ extraction was performed once with all differently processed and dried samples. Astaxanthin analysis of each individual sample was performed in triplicates after each process step. Tests on significant deviations were calculated by mean difference tests with a level of significance of σ = 0.05.

### 2.9. Effort Estimation

An effort estimation was performed to assess the technical and economic feasibility of cell disruption and drying of *Haematococcus pluvialis* biomass. Therefore, the following aspects were evaluated: biomass and astaxanthin recovery rate, cell disruption efficiency (for BM and HPH), residual moisture (for SD and FD), acquisition costs, workload, usability of the equipment, power consumption, hygienic aspects, throughput, sample pre-treatment and scalability. The disruption efficiency refers to comparable disruption grades of algal cells, i.e., after three and one passages in the bead mill and high-pressure homogenizer, respectively. The acquisition costs refer to the costs incurred when purchasing the equipment. The workload includes the time needed for assembly and disassembly, pretreatment/processing of the biomass, as well as cleaning and sanitization steps after usage. Technical data (power, hygienic data, etc.) were obtained from the manufacturer’s data sheets, assuming to use the same devices as those used in the experiments of this study. SD was calculated only based on the pilot-scale spray dryer (Anhydro MS150, SPX FLOW, Charlotte, NC, USA). Only for FD, a scaled device was used for calculation of the technical data (DW-50ND, Drawell Scientific Instrument, Chongqing, China). Usability was evaluated based on the assessment of the complexity of setting up, dismantling, and sanitizing the machines, the simplicity of operation, and what prerequisites, in terms of the level of training of the staff, must be met in order to competently operate the machines. Some process parameters were defined for comparability of the processes: the dry weight of the harvested biomass was defined to be 150 g/L and the working volume was 50 L.

## 3. Results and Discussion

**Figure 1:**
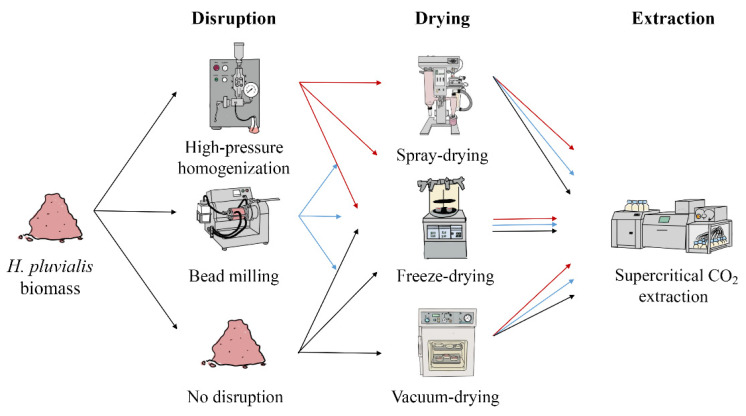
Process steps for the optimization of astaxanthin recovery in the downstream process of *H. pluvialis*.

Astaxanthin extraction from *H. pluvialis* requires disruption and drying as preparation for SC-CO_2_ extraction. To evaluate the efficiency of each step regarding the final astaxanthin yield, different disruption and drying methods were compared. Various combinations of the procedures followed by SC-CO_2_ extraction were assessed to optimize the overall astaxanthin recovery (Figure 1). For all experiments, one initial batch of *H. pluvialis* biomass was used. After each process step, astaxanthin concentration and process parameters such as the disruption rate or residual moisture were determined. Precise astaxanthin concentrations and diastereomer distribution data are given in Table S2. All process steps were evaluated economically. This study aimed to find the optimal combination of process steps for maximum astaxanthin yields at minimal costs.

### 3.1. Disruption of H. pluvialis Biomass

The hypothesis of facilitated astaxanthin extraction by cell disruption was tested by starting the downstream processing with high-pressure homogenization (HPH) or bead milling (BM).

#### 3.1.1. High-Pressure Homogenization

The disruption efficiency of HPH was 81.1 ± 3.7% (*n* = 4) after the first and 92.4 ± 1.6% (*n* = 4) after the second passage at circa 80 MPa. Slightly lower disruption rates of *H. pluvialis* by HPH were observed by Praveenkumar et al. at 68.9 MPa. They showed the decreases in intact cells to be 45%, 20%, and 11% after one, two, and three passages, respectively. However, increases in pressure to 137.9 and 206.8 MPa did not result in higher disintegration rates [67]. Chen et al. observed disruption rates of over 90% at 70 MPa at *H. pluvialis* concentrations of 3% to 5% and one to three HPH passages [68]. A correlation of HPH disruption efficiency with increasing pressure has also been shown for various other microalgae [95], whereas no effect of varying pressure on the disruption rate has been observed in *Nannochloropsis* sp. between 30 and 150 MPa [96]. Higher disruption efficiency with decreasing biomass concentration has been observed for microalgae and *Escherichia coli* cells [97,98]. Kleinig et al. suggested, among other things, the lower viscosity to be the actual cause [97]. Direct comparison of the disruption rate of different *H. pluvialis* batches even at similar conditions may result in significant variances due to differently rigid cell walls. Moreover, the cells in these experiments were frozen prior to HPH, which might have destabilized cell wall integrity.

**Figure 2:**
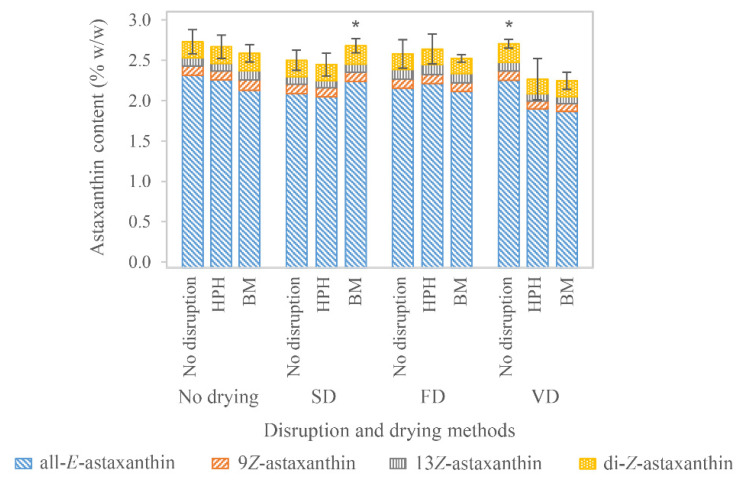
Astaxanthin content in *H. pluvialis* biomass after various disruption and drying processes. Standard deviation is indicated for total astaxanthin. Stars indicate significant differences (*p* < 0.05) from samples dried with the same process. Further significant differences are provided in Table S2.

Homogenization generates heat. In these experiments, the temperature rose from 21 °C to 39.3 °C (*n* = 2) in the first and from 26.7 ± 1.53 °C (*n* = 4) to 36.8 ± 3.6 °C (*n* = 4) in the second pass. This reflects increases of 2.3 °C and 1.3 °C per 10 MPa after the first and second pass, respectively. Increases of 2.2 °C and 2.5 °C per 10 MPa have been reported in other studies as well [99,100]. Heating of the cell suspension might influence astaxanthin stability. At elevated temperatures, carotenoids oxidize and degrade [71,101,102] and isomerize in solvents [89,103,104,105,106,107] but also without [108]. The temperature may locally be well above the mentioned increase, because it emerges by the dissipation of mechanical energy into heat when the suspension collides with the impact ring [109]. Nevertheless, only short exposure to these conditions did not result in astaxanthin deterioration. Total astaxanthin content was 2.73 ± 0.15% *w*/*w* (*n* = 6) before and 2.67 ± 0.14% *w*/*w* (*n* = 12) after two passages of HPH. Before the disruption, astaxanthin was composed of 84.9% all-*E-*astaxanthin, 4.2% 9*Z*-astaxanthin, and 3.61% 13*Z*-astaxanthin, and the di-*Z-*isomers had a proportion of 7.3% (Figure 2). Standard deviations for the single isomers and the number of replicates are provided in Table S2. HPH did not significantly influence the abundance of the geometric isomers. Neither significant astaxanthin degradation nor isomerization were observed compared with the unprocessed biomass; therefore, HPH can be generally assumed as a gentle disruption method. An essential factor might be the very short exposure to unfavorable conditions, which does not significantly harm astaxanthin.

Due to device specifications, the biomass had been diluted prior to HPH. Thus, the subsequent drying processes of high-pressure homogenized cells were performed with the diluted biomass. This dilution step is not desirable on industrial-scale processing because it requires repeated dehydration of the biomass, which is cost- and energy-intensive. It could be prevented on a larger scale, e.g., by high-pressure homogenizers that can cope with higher concentrated biomasses.

#### 3.1.2. Bead Milling

BM of biomass resulted in cell disruption of 32.4 ± 13.3% (*n* = 3), 60.2 ± 20.3% (*n* = 3), and 78.5 ± 11.3% (*n* = 3) after the first, second, and third passage, respectively. Disruption after three cycles of BM was not significantly different from one and two passes of HPH. Cell disruption requires a certain amount of energy. This is often referred to as stress intensity when the specific energy of an individual stress event is considered [110,111]. In agitator bead mills, this energy transfer depends on the applied beads (size and material), their filling level, speed of the agitator discs, the mill geometry, and the viscosity of the sample [112,113,114,115,116]. The throughput and the number of passages influence the dwell time of the product. In turn, the dwell time impacts the duration that a cell is exposed to milling conditions and how often a cell is exposed to a stress event (stress number). Together, stress intensity and number have a major influence on the disintegration rate [111]. Some of the mentioned factors, such as the flow rate, may have contrasting effects on the disruption efficiency, however [117], and should not be oversimplified. In this study, most factors were constant due to a fixed setup. The disruption degree was significantly enhanced by multiple passages, confirming the mentioned influence of the stress number [112,113,116,118,119].

Comparisons with the disruption degree of other milled algae [112,113,114,116,117,118] are complicated due to the variety of influence factors. In addition to those already mentioned, cell characteristics influence their stability [120,121] and play an important role in disintegration [112,116,117]. Nevertheless, 78.5% disrupted cells are a good first approach, considering the rigid cell wall of *H. pluvialis* [57].

The deviation in the disruption rates within the repetitions indicates inhomogeneities, with varying mixing degrees. Consistent disintegration might be improved by using less concentrated algae slurries, because more highly concentrated algae slurries often exhibit higher viscosity and are accompanied by higher energy requirements for pumping [122] and difficulties in mixing [114].

The temperature of the algae paste increased up to 40, 42, and 44 °C after the first, second, and third run, respectively. Cooling of the slurry was performed during the passages, in order to prevent a higher temperature rise. Nevertheless, the increasing viscosity of the paste resulted in slightly elevated temperatures. Generally, the temperature increases throughout the process can be explained by dissipative mechanic energy. It depends on flow rate [115], agitation speed [112], bead filling and size, as well as sample concentration. Temperature is one possibly detrimental factor for astaxanthin stability. However, as long as the average dwell time, and thus, exposure, to higher temperatures is short, the milling process does not necessarily influence astaxanthin negatively, as it can also be concluded from the obtained results. Total astaxanthin content was 2.73 ± 0.15% *w*/*w* (*n* = 6) before and 2.59 ± 0.11% *w*/*w* (*n* = 8) after three passages of milling (Figure 2); however, it was not significantly different. All-*E-*astaxanthin decreased significantly by about 8.1%, whereas 13*Z*-astaxanthin increased by 17.5%. The absolute proportion of the remaining diastereomers did not change significantly. BM has been assessed as a mild process step for recovering proteins, especially when maintaining their proper function is intended [116,118,123]. Therefore, it should not negatively affect pigments such as carotenoids.

### 3.2. Drying of H. pluvialis Biomass

Further dehydration of the biomass is necessary for SC-CO_2_ extraction. Drying biomass is a critical process step as it is often energy-intensive and a potential harm for astaxanthin, because the biomass is already disrupted. The exposure to oxygen is enhanced by high surface areas necessary for evaporation. The latter is also promoted by high temperatures, which supports astaxanthin degradation. Three drying processes were compared regarding their influence on astaxanthin content: 1. Freeze-drying (FD): A method using low temperatures and a protective atmosphere. 2. Vacuum-drying (VD): A similar method operating at slightly elevated temperature and reduced oxygen atmosphere. 3. Spray-drying (SD): A frequently used alternative that utilizes high temperatures and air throughput to dry the biomass. The influence of the previous disruption on the subsequent drying step was investigated for all methods.

#### 3.2.1. Freeze-Drying

FD is widely used to solidify fragile pharmaceutical agents [124] because it is generally considered a mild drying process that maintains the activity of processed substances. FD of *H. pluvialis* resulted in total astaxanthin recoveries of 94.5%, 97.6%, and 98.8% in non-disrupted biomass and samples disrupted by BM and HPH, respectively. However, none of the deviations were considered significant (Figure 2). FD has already been used for algae biomass rich in carotenoids. In *Chlorella vulgaris*, FD was reported superior to hot-air-drying for carotenoid recovery. Nevertheless, only about 43% carotenoid recovery was obtained [125]. FD of *Phaeodactylum tricornutum* did not result in significant carotenoid losses [126]. In fruits and vegetables, recoveries of lycopene (11% to 48%), *β*-carotene (27% and 56%), and lutein (34%) were described after FD [127,128,129], indicating different vulnerabilities of various carotenoids to FD and possibly also surrounding conditions. Zhao et al. observed significantly higher astaxanthin recoveries in freeze-dried *H. pluvialis* extracts than vacuum or otherwise dried samples [130]. Overall, the extensive exclusion of oxygen and low drying temperatures possibly provide protective conditions for astaxanthin. Regarding the diastereomers, some minor differences were observed. All-*E-*astaxanthin decreased 6.9% in the non-disrupted lyophilized samples; however, its proportion to the other isomers was unaffected. Similar results were achieved by Cong et al., who lyophilized astaxanthin-rich krill and reported about a 10% loss of all-*E-*astaxanthin [131]. They also observed a simultaneous decrease in 9*Z-* and 13*Z*-astaxanthin, although their losses were only 6.6% and 3.6%, respectively [131]. In the lyophilized samples of this experiment, a significant decrease in 9*Z*-astaxanthin and also the di-*Z-*isomers was observed in the previously milled samples. In contrast, in non-disrupted and high-pressure homogenized samples, 13*Z-*astaxanthin increased up to 35%, whereas in the former, only its relative proportion changed significantly. This can be attributed to isomerization reactions during the drying process. Although isomerization has been reported to increase at higher temperatures [89,103,104,105,106,107,108], minor changes at lower temperatures are still possible. The texture of all samples was a cake with large pores, which was loose, fluffy, and easy to disintegrate, as it is typical for freeze-dried samples.

#### 3.2.2. Spray-Drying

SD of *H. pluvialis* is a widely applied drying method [53,73]. However, the combination of high temperatures and oxygen exposure, which is enhanced by the surface enlargement during the spray process, increases its potential for astaxanthin degradation. SD also exhibits a variety of setting options. Design of experiment and model estimation and selection were performed to find optimal conditions for drying the specific target. The influence of the obtained parameters was tested for significant influences on yield and astaxanthin degradation. Spray gas pressure, product flow, and inlet temperature were varied at constant biomass concentration and airflow. The amount of the obtained biomass in the collection vessel and its astaxanthin content were measured. The highest biomass amount of 4.93 g, corresponding to 25.1% recovery of the total biomass of the sample, was obtained at a spray gas flow of 400 NL/h, 10% feed rate, and 180 °C. The lowest biomass amount of 0.18 g, corresponding to 0.90% recovery, was obtained at a spray gas flow of 600 NL/h, 10% feed rate, and 180 °C (Table S1). Three different models were estimated to find the best description and optimize the SD conditions. When the parameters were fitted regarding biomass yield, the quadratic model, including the interaction terms, described the results best when comparing the adjusted coefficients of determination (Table 1).

The model does not allow accurate assessment of the single parameters, but it shows the importance of the interaction terms (*p* < 0.05). Interactions between the different settings in SD have been assumed [132,133,134]. This is plausible because a higher feed rate reduces temperature as more water evaporates. A higher feed rate also influences the spray behavior, and the difference in droplet size probably also influences the temperature. Consequently, the quadratic model, including the interaction terms, was chosen to describe SD behavior best regarding biomass yield. Generally, the highest temperature (180 °C) was assessed to be optimal in terms of yield in all models. Such positive effects were also observed in the SD of other products [132,135] and differently concentrated microalgae samples. In the latter, the highest yields were reported at 220 °C compared with 170 °C and 120 °C [136]. Higher drying temperatures result in faster evaporation and likely less biomass sticking to the walls of the device and thus higher proportions reaching the collection vessel.

Lower spray gas flows have been considered to improve product yields in SD by increasing particle size and facilitating capturing by centrifugal forces in the cyclone [133]. A similar correlation was confirmed here, as the lowest volume flow was considered optimal in all models, even though a significant influence of this parameter could only be observed in the linear model.

A higher product feed rate has been described to result in smaller yields; however, at lower temperatures than used in these experiments [134]. A higher feed rate will eventually result in lower yields when the drying capacity of the spray-dryer is overloaded. The feed rate range in these experiments was limited. Thus, this tendency is not reflected by the models. Again, the data from the quadratic model, including the interaction terms, were considered most reliable. Thus, a medium feed rate of 10.4%, 5.8 mL/min, was assumed optimal.

Astaxanthin content decreased only slightly after drying in most samples (Table S2). Before drying, total astaxanthin amount of the biomass was 1.32 ± 0.03% *w*/*w* (*n* = 3). After drying, the highest total astaxanthin contents of 1.22% *w*/*w*, obtained at a spray flow of 400 NL/h, 10 to 15% feed rate, and 160 to 180 °C, were not very different from the astaxanthin content before drying. The lowest astaxanthin content of 0.91 ± 0.03% *w*/*w* (*n* = 3) was obtained at a spray flow of 600 NL/h, 10% feed rate, and 180 °C. However, most of the astaxanthin proportions did not differ from each other, impeding proper model evaluation (Table S3). These findings indicate an almost negligible influence of many SD conditions on astaxanthin content. No effect on isomer composition was observed and is in agreement with Leach et al., who investigated *β*-carotene in microencapsulated and spray-dried *Dunaliella salina* [137].

SD at the previously considered optimal parameters (10% feed rate, 400 NL/h spray flow, 180 °C inlet temperature) resulted in 1.14 ± 0.07% *w*/*w* (*n* = 3) total astaxanthin. This was only slightly different from the maximum result. Therefore, the parameters optimizing the biomass yield were considered best for the subsequent experiments. They were applied once more to evaluate the influence of water content in the sample by drying 200 mL of the same biomass diluted to 5% *w*/*v* dry mass and 200 mL of another batch of biomass at 20% *w*/*v* dry mass. Results were 2.58 g and 6.13 g biomass in the collection vessel, corresponding to 24.6% and 15.2% recovery. It might be concluded that higher dry matter content negatively influences the yield in SD. However, a converse correlation was observed by the manufacturer of the spray-dryer, who reported higher yields in dried microalgae samples with a concentration of 17% *w*/*w* than 9% *w*/*w* and assumed the bigger particles beneficial in separation [136]. Bigger droplets do not dry as fast as smaller ones, and might also be more likely to stick to the walls of the device instead of being transported into the collection container, which could be the reason for the lower yield in these experiments. Nevertheless, a recovery of 15.2% was acceptable for the planned experiments and meant a sufficient absolute biomass quantity for subsequent downstream processing. Further optimization was not performed. However, on an industrial scale, the biomass recovery after drying is crucial and has to be enhanced.

Using the optimal parameters obtained from the design of experiments, spray-drying with the same biomass as used in the other drying experiments was performed. Total astaxanthin recovered after SD decreased to 91.6% and 91.7% in non-disrupted biomass and samples disrupted by HPH, respectively. This loss was mainly attributed to a decrease in all-*E-*astaxanthin. The losses observed after SD were higher than after FD. Ahmed et al. described an even more pronounced effect of 29% less astaxanthin in spray-dried than in freeze-dried *H. pluvialis* samples immediately after drying [71]. Other studies showed a significant decrease in carotenoids in spray-dried *Phaeodactylum tricornutum* [126] and *Rhodotorula glutinis* [138]. Total astaxanthin content in the milled biomass stayed constant after SD at the laboratory scale as well as the pilot scale. In the latter, total astaxanthin content in non-disrupted samples was 2.15% *w*/*w* and decreased to 2.01%, 2.00%, and 1.88% *w*/*w* in samples milled one, two, and three times, respectively. Spray-dried samples exhibited 1.85% *w*/*w* total astaxanthin, which was insignificantly different from all milled samples but significantly lower than in the non-disrupted biomass. All diastereomers were unaffected by SD at the pilot scale, whereas at the laboratory scale, a decrease in 9*Z-* and 13*Z*-astaxanthin was accompanied by an increase in all-*E-*astaxanthin, indicating isomerization reactions. No obvious isomerization has been reported in *β*-carotene during SD [137]. Generally, the deteriorating effect of exposure to 180 °C at the inlet was not as severe as expected. This was confirmed by other studies, which even observed higher astaxanthin recovery with increasing SD temperatures [72,102]. However, the reverse correlation was reported for *β*-carotene from *Dunaliella salina*. Here, an increase to 265 °C in inlet and 120 °C in outlet temperature during SD caused a reduced recovery of *β*-carotene, which was only diminished by a lower feed concentration or microencapsulation [137]. Such effects of feed concentration were not observed in these experiments. Overall, the rapid drying process and product cooling by evaporation reduced carotenoid oxidation and degradation, as also assumed by Leach et al. [137]. Thus, SD is not as devastating as expected regarding the astaxanthin content and remains an option for the downstream processing of *H. pluvialis.*

Spray-dried biomass mostly had a powdery texture. In previously high-pressure homogenized samples, smaller spheres of 1 to 4 mm in diameter were formed. They had a loose coherence and easily disintegrated. They were also observed in two milled samples to a smaller extent. Biomass recovery throughout the spray-dryer was similar for non-disrupted and milled biomass. The biomass recovered in the collection vessel was not significantly different in all experiments. It was 20.63 ± 0.49% (*n* = 3), 21.49 ± 6.28% (*n* = 3) and 25.42 ± 3.24% (*n* = 3) for non-disrupted, high-pressure homogenized and milled samples, respectively. However, drying of high-pressure homogenized samples resulted in a heavier loaded cyclone and a less loaded spray tower compared with milled and non-disrupted biomass. The loading for non-disrupted and milled samples was highly similar; thus, the disruption and accompanying changes in viscosity seemed not to influence the drying and adhesion behavior in the device. More likely, the dilution of the biomass affects it, because this was the only significantly different parameter. For optimal total astaxanthin recovery, biomass yield is equally important. In FD and VD, biomass losses to the walls of the vessels are negligible. However, the surface to which biomass can stick is much larger in SD, and although biomass can be scraped off after drying, greater biomass and astaxanthin losses are very likely; material that is stuck to the wall of the spray tower is exposed to higher temperatures over a longer time, and astaxanthin is more likely to degrade here. These issues have to be addressed in the optimization of SD processes, as well as in economic evaluations. The use of modifiers or (micro)encapsulation with various wall materials has been reported to affect carotenoid losses, yields, and antioxidant activity during drying and/or subsequent storage beneficially [68,139,140].

#### 3.2.3. Vacuum-Drying

VD has been reported to affect plant and algae bioactive compounds less detrimentally than drying with heat steps [141,142]. The texture of the dried biomass was very dense. It was a rigid and even rubbery cake that was hardly disintegrable. Total astaxanthin recovery after VD was 86.8% and 84.9% in samples disrupted by BM and HPH, respectively. Astaxanthin in non-disrupted biomass was not significantly affected by VD. The extensive exclusion of oxygen and only slightly elevated temperatures were intended to reduce the oxidation of astaxanthin during VD. However, this only applied to the non-disrupted samples. Again, the intact, rigid cell wall of *H. pluvialis* probably protects astaxanthin from environmental influences, resulting in lower degradation.

The diastereomers were generally more affected in the disrupted samples. In milled biomass, the proportion of 9*Z-* and 13*Z*-astaxanthin decreased, whereas in high-pressure homogenized samples, the proportion of 13*Z*-astaxanthin increased significantly. In non-disrupted biomass, the proportional decrease in all-*E-*astaxanthin was accompanied by an increase in the di-*Z-*isomers.

Comparing all the different drying processes, higher total astaxanthin recovery was observed in non-disrupted and vacuum-dried samples than in non-disrupted and freeze- or spray-dried samples. This may be because of the more compact sample during VD, reducing the surface area and thus possible reactions of astaxanthin with residual environmental oxygen. However, disrupted biomass yielded the lowest recoveries in VD. Here, the longer and direct exposure of astaxanthin to the drying conditions might cause enhanced degradation. Overall, the best results were obtained from milled and spray-dried biomass, whereas milled or high-pressure homogenized freeze-dried biomass was equally promising. These findings indicate that BM and HPH are suitable disintegration methods, and SD and FD are both appropriate for drying disrupted *H. pluvialis* biomass without severe astaxanthin losses. Astaxanthin recoveries obtained after the different combinations of disruption and drying methods are shown in Figure 2.

### 3.3. Supercritical CO_2_ Extraction of Astaxanthin

**Figure 3:**
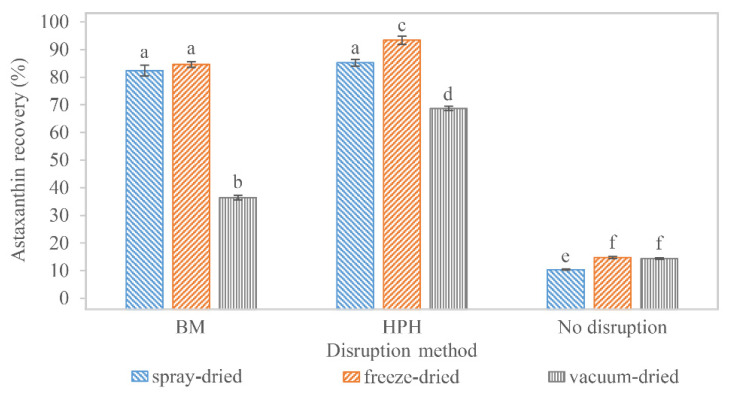
Astaxanthin recovery in supercritical fluid extracts of differently disrupted and dried *H. pluvialis* biomass. Significant differences (*p* < 0.05) of the samples are indicated by different letters.

Astaxanthin was still bound to the disrupted biomass of *H. pluvialis* after the drying processes. SC-CO_2_ extraction was performed to separate and concentrate astaxanthin. Total astaxanthin recovery was lowest in the SC-CO_2_ extracts of non-disrupted *H. pluvialis* biomass. Here, 10.40 ± 0.23% (*n* = 3), 14.72 ± 0.37% (*n* = 3), and 14.32 ± 0.28% (*n* = 3) of total astaxanthin were extracted from spray-dried, lyophilized, and vacuum-dried biomass, respectively. An overview of all recoveries is shown in Figure 3. Standard deviations of samples were evaluated for triplets or quartets. Diastereomer composition can be found in Table S2. The low recovery can be explained by the poor extractability of intact *H. pluvialis* cells. Generally, mass transfer in SC-CO_2_ extraction of plant material is improved when the transport resistance across cell walls and/or membranes is reduced [143]. Here, SC-CO_2_ could hardly penetrate the rigid cell wall, and solution and transfer of astaxanthin were impeded. This suggests that cell disruption is a key component in the downstream processing of *H. pluvialis*. Comparing only these non-disrupted samples, extractability from vacuum-dried and lyophilized biomass was similar, whereas it was significantly lower from spray-dried biomass. This might be because of the vacuum damaging some of the cells and thus facilitating extractability slightly.

Extracts of milled samples that were spray-dried, lyophilized and vacuum-dried reached recoveries of 82.41 ± 1.96% (*n* = 3), 84.61 ± 1.03% (*n* = 3), and 36.45 ± 0.84% (*n* = 3) total astaxanthin, respectively, as compared with their counterparts prior to extraction. No significant difference in extractability was observed in samples that had been lyophilized or spray-dried. The significantly lower extractability of vacuum-dried samples was possibly caused by the texture of the biomass. Different models describe mass transfer in SC-CO_2_ extraction. Very simplified, molecules at the solid to supercritical fluid interface are extracted first. If these are washed out, mass transfer is additionally determined by the solute diffusivity within the solid (through pores) to the interface [143,144]. The accessibility of astaxanthin in this step was probably easier in spray-dried and lyophilized biomasses, which had a porous texture with a high surface, whereas vacuum-dried biomass was very dense and solid.

Total astaxanthin yields in extracts of high-pressure homogenized samples were 85.25 ± 1.21 (*n* = 4), 93.38 ± 1.49 (*n* = 3), and 68.68 ± 0.78% (*n* = 3) of previously spray-dried, lyophilized, and vacuum-dried samples, respectively. The lower extractability of the vacuum-dried samples can be explained similarly to the milled samples. The higher extractability of lyophilized samples might be due to the effect of breaking residual intact cells by the vacuum and an even looser texture than that obtained after SD. The higher extractability of (lyophilized and vacuum-dried) high-pressure homogenized biomass compared to their bead milled counterparts might be because of their higher disruption degree, and thus, easier accessibility of astaxanthin during SC-CO_2_ extraction.

Altogether, the extracts of high-pressure homogenized samples that were freeze-dried yielded the highest astaxanthin recoveries, closely followed by high-pressure homogenized samples that were spray-dried and milled samples that were either spray- or freeze-dried. Between the latter, no significant difference was observed. Comprehensive literature on optimal SC-CO_2_ extraction conditions for astaxanthin recovery from *H. pluvialis* is available, and the influences of pressure, temperature, flow rate, and co-solvents have been discussed [78,79,80,81,82,84,85,86]. Nevertheless, these experiments showed that pre-processing of the samples was equally essential. Positive effects of cell disruption on carotenoid recovery have already been indicated in general and in SC-CO_2_ extraction [119,145]. Nobre et al. reported total carotenoid recoveries of 91.8% in lyophilized, crushed (vibratory mill), and SC-CO_2_-extracted *H. pluvialis* [54]. This is similar to the results shown here for high-pressure homogenized cells. Nobre et al. and Valderrama et al. also stressed the impact of the crushing intensity and SC-CO_2_ extraction conditions on extractability [54,55]. This facilitated mass transfer of astaxanthin into SC-CO_2_ due to prior cell disruption could also be confirmed. Moreover, drying affects extractability if it enhances the coherence of the biomass, as observed in the vacuum-dried samples. The observed astaxanthin recoveries were generally correlated with the extract yields.

Total astaxanthin was concentrated 1.35- to 4.15-fold in evaporated SC-CO_2_ extracts compared with the concentration in the biomass of the corresponding samples. Thereby, the concentration of all diastereomers increased significantly in all extracts except for one (non-disrupted and spray-dried). However, the composition of the different isomers changed. In all extracts, the proportion of all-*E-*astaxanthin increased, whereas the proportion of other diastereomers decreased (Table S2). This effect was significant for the majority of diastereomers and samples. Álvarez et al. reported even higher all-*E-* and 9*Z*-astaxanthin proportions in their SC-CO_2_ extracts. However, they did not include the di-*Z-* isomers [78]. Other studies indicated higher *Z*/*E-*ratios of various carotenoids in supercritical fluid extracts due to their higher solubility [145,146,147]. Selective extractability is also dependent on SC-CO_2_ extraction parameters [148,149] and isomerization during extraction [150], but was not confirmed in the SC-CO_2_ extraction of *H. pluvialis* at different temperatures and pressures [78]. Moreover, isomerization in the later analysis cannot be excluded, and might lead to higher 9*Z-* and di-*Z-*astaxanthin isomer shares [88]. Although the exact influence of various process conditions on isomer ratios was not precisely evaluated, SC-CO_2_ extraction likely affects the composition of astaxanthin diastereomers in the resulting extracts.

### 3.4. Overall Astaxanthin Yield

Regarding the whole process, maximum astaxanthin yield was recovered after SC-CO_2_ extraction when using HPH combined with FD (85.4%) or SD (81.6%). Similar results were obtained using BM and SD (79.0%) or FD (78.1%). VD did not result in good recoveries for all samples, possibly because of the discussed change in sample texture, i.e., high-pressure homogenized (60.5%), milled (30.9%), and non-disrupted (14.0%), respectively. No good recovery rates were obtained from non-disrupted samples, regardless of the drying process (FD 13.7% and SD 9.4%). It can be concluded that SC-CO_2_ extraction yielded the best results when the biomass was disintegrated before extraction, with as few cells remaining intact as possible. VD resulted in textural changes in the biomass, which deteriorated the efficiency of SC-CO_2_ extraction. However, BM and HPH combined with SD and FD and subsequent SC-CO_2_ extraction of *H. pluvialis* biomass resulted in similarly high astaxanthin yields. Thus, all these processes might be applicable in downstream processing regarding astaxanthin maximization (Table 2), but biomass yields, acquisition, labor, and operating costs also have to be considered.

### 3.5. Effort Estimation

An effort estimation to assess technical and economic feasibility of cell disruption and drying of *Haematococcus pluvialis* biomass was performed. Such evaluations of the practical application of the proposed methods and their technical and economic feasibility are crucial in setting up a commercial realization of an astaxanthin production process. We demonstrated astaxanthin losses in every combination of the methods for cell disruption and drying. Some of these losses can be attributed insufficient cell disruption. Hardly any astaxanthin was extracted from unbroken *H. pluvialis* cells. Hence, choosing the right disruption method is essential for the profitability of commercial astaxanthin productions. The difference in the astaxanthin yields between the individual full processes (disruption and subsequent drying) was rather small. Accordingly, no clear recommendation for a specific process can be based on the astaxanthin recovery rates only. Other evaluation criteria need to be considered (Table 3).

For cell disruption, both methods are rather similar with respect to the effort: the acquisition costs for a pilot-scale bead mill are around EUR 70,000. Here, an actual throughput of 3.3 L/h was considered, because three passages of the biomass are necessary for adequate disruption rates. A comparable HPH system costs around EUR 40,000. Both methods have a similar throughput and are scalable for commercial processes. User-friendliness is also given as both devices are easy to use. The cleaning times and the respective cleaning effort are also similar. The power consumption of both devices is also the same, as well as the performance at equal throughput. However, there are also significant differences between the two approaches. The BM requires three runs to achieve a high level of cell disruption (~78%). HPH only needs one passage for the same level of disruption (~81%). The second passage increases the disruption rate to ~92%. Therefore, the disruption efficiency of the HPH can be regarded as higher than that of the BM. HPH can be a very time-consuming process. The material had to be processed first to remove clumps or other coarse pieces (biofilm residues), because HPH is prone to clogging by impurities or non-uniform particle sizes. This process can be time-consuming, depending on the amount and the condition of the biomass, and might be a major bottleneck to the HPH of *H. pluvialis* cells.

For HPH, the biomass density should not be higher than 50 g/L. Therefore, the biomass had to be diluted first. Assuming a biomass density after harvesting of 150 g/L, a dilution factor of three is needed. This increases a working volume of 50 to 150 L, causing a subsequent three-fold increase in disruption time for HPH compared with BM. That also increases costs for the following drying step. Assuming that the BM needs three runs and the HPH needs one run, the duration of the cell disruption is more than three times higher with HPH compared with BM. Accordingly, the overall working time and thereby the overall labor costs rise. Thus, BM is more efficient regarding working time and energetic costs. The cleaning procedures of both devices were a bit different. For cleaning the HPH, water was pumped through the system followed by disinfecting agents. The cleaning of the BM was a bit more complex due to the disassembling and reattachment of all product contacting parts, including the beads that had to be cleaned separately. In contrast to the cleaning procedure of HPH, the cleaning step for the BM took longer because every part was cleaned manually. However, this can result in a higher degree of cleanliness. Although the initial costs are higher, and the disruption efficiencies can be lower, we recommend BM for cell disruption on a larger scale because the overall working time, labor costs, as well as the overall power consumption are lower compared with a process that uses HPH. It is also important to consider that BM has many interacting parameters and still further optimization potential.

Regarding astaxanthin recovery, the drying process was most efficient using SD or FD. VD resulted in higher astaxanthin degradation and lower extractability in the SC-CO_2_ extraction, and was therefore not included in the effort estimation. Both SD and FD showed similar content of residual moisture in the biomass after drying (<9%). Each process is already used for largescale production, which proves the scalability, e.g., FD is used to produce plant-based foods [151,152] and SD is used to produce a variety of products in the food and beverage industry, e.g., milk powder, soy protein, etc. [153]. Using FD, the process of drying can take several hours for one batch. In contrast, with SD, the material is dried immediately when it passes through the machine. However, because the operation of the FD is very simple and the device works without the necessity of constant supervision, workload and labor costs are reduced compared with other approaches. Nevertheless, FD cannot be operated continuously. This means that depending on the volume of biomass and the size of the FD, several cycles might be necessary. This increases the workload and the overall drying time. In that case, the disrupted algal biomass must be stored temporarily, implicating additional time and space requirements. The cleaning effort for SD was higher because the entire interior as well as the pipes were in contact with the biomass and needed to be cleaned. The biomass accumulated in the machine during the drying process was collected entirely in the pilot-scale process and only from the collection vessel in the laboratory-scale experiments. This is why the losses of biomass during pilot-scale SD were lower than at the laboratory scale and assumed similar to FD. With FD, only the trays that contained the biomass needed to be cleaned. Therefore, the cleaning time was approximately 3 times longer with SD. The main disadvantage of FD is power consumption for freezing, sublimation, condensation, and creating a vacuum in comparison to heat-drying processes [154]. Thus, the power consumption of FD was calculated to be 11 times higher in comparison with SD. Similar results were obtained in a study that compared the costs and energy demand of SD and FD of 100 kg *H. pluvialis* biomass. Here, FD has been shown to be more than 7 times more energy-consuming than SD [71]. As shown, astaxanthin recovery rates were similar after FD and SD. FD might be more suitable regarding the stability and shelf life of astaxanthin [71]; however, this is negligible when the samples are quickly further processed. The high temperatures of up to 180 °C inside the drying vessel and an outlet temperature of 80–90 °C that were applied for SD led to a high degree of sanitization in the resulting product. In cases of potential presence of bacteria and fungi in the biomass, this step improves the product quality and food safety. SD is not a sterilizing process, even for non-spore-forming organisms, but will substantially reduce bacterial populations in products [155,156]. If the machines are regularly maintained and fully functional, no high level of education or specialist knowledge is required to operate them. Although the initial costs are higher, for a larger scale, we recommend using a spray-dryer for biomass drying because the overall working time, labor costs, and overall power consumption are lower compared with a process that uses FD.

Publications focusing on the larger-scale production of astaxanthin most often used a disruption step and SD. Some describe a similar set up of BM and SD followed by SC-CO_2_ [53,157]. Li et al. changed their order and used SD prior to an airflow pulverizer for disintegration, and did not include an additional extraction step [52]. Péréz-Lopez et al. only used SD prior to SC-CO_2_ extraction, but omitted a disintegration step [87]. Based on the results of this article, we highly recommend a disruption step for easier extractability, and thus higher astaxanthin recoveries. For the complete downstream process, BM followed by SD of *H. pluvialis* biomass was estimated as optimal pretreatment for SC-CO_2_ extraction when additionally considering economic parameters.

## 4. Conclusions

Various downstream process steps were combined in all possible combinations and the astaxanthin recovery was assessed. A disruption of *H. pluvialis* was necessary to facilitate SC-CO_2_ extraction. Neither BM nor HPH influenced the total astaxanthin content significantly, thus only their ability to disrupt the biomass efficiently had a possible influence in the later extraction. Here, HPH resulted in a 14% higher disruption degree and lower variance. Favorable SD conditions concerning astaxanthin recovery and biomass yield were determined with design of experiments and standard least squares regression. The impacts of SD and FD on the astaxanthin content was generally low or even negligible; only VD resulted in a considerable decrease of 13% to 15% total astaxanthin and a change in the texture of the samples, which impeded SC-CO_2_ extraction. Considering the whole process chain, the highest astaxanthin recovery of 85.4% was achieved using HPH and FD; however, all process combinations involving a disruption process and SD or FD resulted in similarly good results. FD and HPH are quite expensive. Therefore, BM was combined with spray-drying on larger scale. The results concerning the astaxanthin recovery were comparable to those on laboratory scale. However, also economic parameters have to be taken into consideration. The feasibility studies showed that BM combined with SD can also be recommended in an economic context.

## Figures and Tables

**Table 1:** Estimated model coefficients, *p*-values, and optimized parameters regarding maximal biomass yield. Significant *p*-values (σ = 0.05) are highlighted in bold.

	Linear Model		Quadratic Model		Quadratic Model + Interactions	
	Coefficient	*p*-Value	Coefficient	*p*-Value	Coefficient	*p*-Value
A (Intercept)	7.175		27.5392		−23.6551	
b_1_ (S ^a^)	−0.0178	**0.0006**	−0.0848	0.212	−0.0349	0.48853
b_2_ (F ^b^)	0.0294	0.2422	−0.0326	0.9482	−6.4457	**0.01817**
b_3_ (T ^c^)	0.0196	0.7315	−0.0295	0.9082	0.7736	0.07361
c_1_ (S^2^)			0.0001	0.3117	0.0001	0.06927
c_2_ (F^2^)			0.0031	0.8963	−0.0661	**0.04148**
c_3_ (T^2^)			0.0001	0.859	−0.002	0.08029
d_12_ (S*F)					0.0044	**0.02337**
d_13_ (S*T)					−0.0008	**0.04314**
d_23_ (F*T)					0.0337	**0.02003**
R^2^	0.7739		0.8085		0.9612	
R^2^ adjusted	0.7061		0.6444		0.8739	
Optimized parameters						
F	15		15		10.4	
S	400		400		400	
T	180		180		180	

^a^ S, spray gas flow (NL/h); ^b^ F, product feed rate (%); ^c^ T, inlet temperature (°C).

**Table 2:** Total astaxanthin recovery after SC-CO_2_ extraction compared with the initial astaxanthin content of the sample, depending on the previous disruption and drying methods.

Disruption	Drying	Recovery (%)
No disruption	Freeze-drying	13.7 ± 0.35
No disruption	Spray-drying	9.4 ± 0.21
No disruption	Vacuum-drying	14.0 ± 0.27
Milling	Freeze-drying	78.1 ± 0.95
Milling	Spray-drying	79.0 ± 1.88
Milling	Vacuum-drying	30.9 ± 0.71
High-pressure homogenization	Freeze-drying	85.4 ± 1.36
High-pressure homogenization	Spray-drying	81.6 ± 1.56
High-pressure homogenization	Vacuum-drying	60.5 ± 0.69

**Table 3:** Comparison of technical data of the used disruption and drying devices. n.a., not applicable.

	Cell Disruption		Biomass Drying	
	Bead Mill	High-Pressure Homogenizer	Spray-Dryer	Freeze-Dryer
Throughput	10 L/h	3 L/h	5 L/h	0.4 L/h
Power consumption	4 kW	4 kW	5 kW	4.5 kW
Disruption efficiency	high	high	n.a.	n.a.
Residual moisture	n.a.	n.a.	<9%	<9%
Time for setup	<0.5 h	<0.5 h	1 h	<0.5 h
Time for processing	15 h	50 h	10 h ^a^/30 h ^b^	120 h ^a^/360 h ^b^
Cleaning time	1 h	<1 h	1–2 h	<0.5 h
Cleaning procedure	simple	very simple	difficult	very simple
Overall workload	32 h	61 h	12 h	2 h
Product sanitization	n.a.	n.a.	very high	high
Usability	simple	simple	moderate	simple
Scalability	yes	yes	yes	yes
Acquisition costs	EUR 70,000	EUR 40,000	EUR 250,000	EUR 27,000

^a^ when BM is used for cell disruption; ^b^ when HPH is used for cell disruption.

## Supplementary Materials

The following supporting information can be downloaded at: https://www.mdpi.com/article/10.3390/foods11091352/s1, Table S1: Overview of process parameters and results of the conducted experiments for model regression and evaluation in spray-drying, Table S2: Content and proportion of astaxanthin and its diastereomers after various processing steps, Table S3: Estimated model coefficients, p-values and optimized parameters regarding maximal astaxanthin yield in spray-drying.
