# Supplementary material for: Optimization of Astaxanthin Recovery in the Downstream Process of Haematococcus pluvialis
Source: Foods. 2022 May 6;11(9):1352. doi: 10.3390/foods11091352 (PMC9105871; doi:10.3390/foods11091352)
Supplement: Supplementary file 1 [file foods-11-01352-s001.zip › foods-1702049-supplementary.pdf]

Supplementary Information for **Optimization of Astaxanthin Recovery in the Downstream Process of *Haematococcus pluvialis***

Inga K. Koopmann [1], Simone Möller [1,2], Clemens Elle [2], Stefan Hindersin [2], Annemarie Kramer [1,*] and Antje Labes [1,*]

Table S1: Overview of process parameters and results of the conducted experiments for model regression and evaluation in spray-drying.

	Input					Output					
	Inlet temperature	Spray flow	Feed rate	Cooling	Biomass conc.	*H. pluvialis* biomass recovery		Astaxanthin content			Outlet temperature
								per biomass		recovery	
Run	(°C)	(NL/h)	(%)	-	(g/L)	(g)	(%)	(% w/w)	SD[a)]	(%)	°C
Before					99.4			1.32	0.03	-	
1	140	500	5	no	99.4	1.2	6.04	1.17	0.04	88.6	99
2	160	500	10	no	99.4	0.56	2.82	1.08	0.07	81.8	98
3	160	600	15	no	99.4	1.03	5.18	1.06	0.05	80.3	80
4	180	400	10	no	99.4	4.6	23.01	1.14	0.07	86.4	112
4.2	180	400	10	no	99.4	4.93	25.1	-			134
5	140	400	5	no	99.4	3.78	19.54	1.21	0.003	91.7	95
6	160	400	5	no	99.4	3.92	19.51	1.1	0.04	83.3	124
7	120	500	5	no	99.4	0.43	2.09	1.1	0.03	83.3	85
8	180	600	10	no	99.4	0.18	0.9	0.91	0.03	68.9	111
9	180	500	10	no	99.4	2.31	12.02	1.15	0.15	87.1	122
10	180	400	5	no	99.4	2.81	14.12	1.16	0.04	87.9	134
11	180	400	15	no	99.4	3.68	18.5	1.22	0.09	92.4	115
12	160	400	10	no	99.4	2.36	11.84	1.22	0.1	92.4	107
13	180	500	15	no	99.4	1.82	9.83	1.15	0.08	87.1	114
14	180	400	10	yes	99.4	4.74	23.41	-		-	123
15	180	400	10	yes	49.7	2.58	24.62	-		-	121
16	180	400	10	yes	198.7	6.13	15.2	-		-	122

[a)]SD = Standard deviation

Table S2: Content and proportion of astaxanthin and its diastereomers after various processing steps. Colors indicate comparison of samples.

		Total astaxanthin					all-*E*-astaxanthin						9*Z*-astaxanthin						13*Z*-astaxanthin						di-*Z*-astaxanthin					
**Process Step**	n[a)]	Ax/*H.p.*[b)]	SD[c)]	Sig[d)]	Sig	Sig	Ax/*H.p.*	SD	Sig	Prop.[e)]	SD	Sig	Ax/*H.p.*	SD	Sig	Prop.	SD	Sig	Ax/*H.p.*	SD	Sig	Prop.	Stab	Sig	Ax/*H.p.*	SD	Sig	Prop.	Stab	Sig
**Analytical scale**																														
Non-disrupted	6	2.73	0.15	-	-	a	2.31	0.09	-	84.88	2.05	-	0.12	0.03	-	4.21	0.78	-	0.10	0.02	-	3.61	0.43	-	0.20	0.04	-	7.30	0.92	-
BM[f)] (1x)	10	2.68	0.15	e	-	-	2.20	0.14	e	82.09	1.92	*	0.13	0.02	e	4.89	0.66	e	0.12	0.02	*	4.49	0.59	*	0.23	0.03	e	8.53	1.02	*
BM (2x)	9	2.62	0.11	e	-	-	2.14	0.11	*	81.73	1.60	*	0.13	0.01	e	4.95	0.62	e	0.12	0.01	*	4.55	0.39	*	0.23	0.02	e	8.78	0.85	*
BM (3x)	8	2.59	0.11	e	-	a	2.13	0.12	*	82.23	1.46	*	0.13	0.01	e	4.90	0.57	e	0.12	0.01	*	4.49	0.31	*	0.22	0.02	e	8.38	0.85	*
BM - SD[g)]	9	2.68	0.09	e	a	a	2.24	0.08	*	83.40	0.65	*	0.12	0.01	*	4.38	0.19	e	0.10	0.00	*	3.72	0.18	*	0.23	0.01	e	8.50	0.31	e
BM - FD[h)]	9	2.52	0.05	e	b	a	2.11	0.05	e	83.74	0.74	*	0.11	0.01	*	4.22	0.26	*	0.12	0.01	e	4.63	0.31	e	0.19	0.01	*	7.41	0.45	*
BM - VD[i)]	9	2.25	0.11	*	c	a	1.87	0.09	*	83.05	0.38	e	0.10	0.01	*	4.29	0.21	*	0.09	0.00	*	3.91	0.17	*	0.20	0.01	*	8.75	0.29	e
BM - SD - SC-CO_2[j)]	3	8.33	0.20	*	-	-	7.18	0.18	*	86.26	0.16	*	0.28	0.02	*	3.36	0.27	*	0.28	0.01	*	3.37	0.08	*	0.58	0.02	*	7.01	0.09	*
BM - FD - SC-CO_2	3	8.78	0.11	*	-	-	7.58	0.08	*	86.30	0.18	*	0.29	0.02	*	3.26	0.29	*	0.29	0.01	*	3.31	0.11	*	0.63	0.04	*	7.13	0.34	e
BM - VD - SC-CO_2	3	6.90	0.16	*	-	-	5.91	0.14	*	85.68	0.04	*	0.22	0.00	*	3.23	0.07	*	0.22	0.01	*	3.23	0.09	*	0.54	0.01	*	7.86	0.10	*
Non-disrupted	6	2.73	0.15	-	-	-	2.31	0.09	-	84.88	2.05	-	0.12	0.03	-	4.21	0.78	-	0.10	0.02	-	3.61	0.43	-	0.20	0.04	-	7.30	0.92	-
HPH[k)] (1x)	12	2.76	0.06	e	-	-	2.34	0.05	e	84.77	1.04	e	0.12	0.01	e	4.25	0.27	e	0.09	0.01	e	3.41	0.21	e	0.21	0.02	e	7.57	0.64	e
HPH (2x)	12	2.67	0.14	e	-	a	2.26	0.12	e	84.64	1.10	e	0.11	0.01	e	4.12	0.28	e	0.09	0.01	e	3.50	0.30	e	0.21	0.02	e	7.74	0.68	e
HPH - SD	9	2.45	0.14	*	b	b	2.05	0.12	*	83.69	0.56	*	0.11	0.01	e	4.57	0.33	*	0.09	0.00	e	3.66	0.20	e	0.20	0.01	e	8.08	0.27	e
HPH - FD	9	2.64	0.19	e	a	a	2.21	0.15	e	83.85	1.16	e	0.11	0.02	e	4.15	0.41	e	0.13	0.02	*	4.76	0.45	*	0.19	0.02	e	7.24	0.51	e
HPH - VD	9	2.27	0.25	*	b	a	1.90	0.20	*	83.78	0.63	e	0.10	0.01	*	4.26	0.20	e	0.09	0.01	e	3.96	0.23	*	0.18	0.03	*	8.00	0.46	e
HPH - SD - SC-CO_2	4	10.15	0.14	*	-	-	8.68	0.17	*	85.54	0.43	*	0.38	0.06	*	3.73	0.62	e	0.35	0.01	*	3.46	0.15	e	0.74	0.03	*	7.27	0.24	*
HPH - FD - SC-CO_2	3	8.83	0.09	*	-	-	7.50	0.10	*	85.04	1.39	e	0.36	0.04	*	4.12	0.44	e	0.30	0.03	*	3.38	0.31	*	0.66	0.06	*	7.46	0.65	e
HPH - VD - SC-CO_2	3	7.17	0.08	*	-	-	6.11	0.07	*	85.22	0.41	*	0.26	0.02	*	3.56	0.33	*	0.24	0.01	*	3.36	0.08	*	0.56	0.04	*	7.85	0.45	e
Non-disrupted	6	2.73	0.15	-	-	-	2.31	0.09	-	84.88	2.05	-	0.12	0.03	-	4.21	0.78	-	0.10	0.02	-	3.61	0.43	-	0.20	0.04	-	7.30	0.92	-
ND[l)] - SD	9	2.50	0.12	*	b	b	2.09	0.08	*	83.67	3.65	e	0.12	0.03	e	4.57	1.06	e	0.09	0.02	e	3.72	0.78	e	0.20	0.05	e	8.04	1.82	e
ND - FD	9	2.58	0.18	e	ab	a	2.16	0.13	*	83.66	1.36	e	0.11	0.02	e	4.22	0.49	e	0.12	0.02	e	4.58	0.50	*	0.20	0.03	e	7.55	0.53	e
ND - VD	9	2.70	0.05	e	a	b	2.25	0.05	e	83.27	0.64	*	0.11	0.01	e	4.25	0.19	e	0.11	0.01	e	3.92	0.25	e	0.23	0.01	*	8.56	0.39	*
ND - SD - SC-CO_2	3	3.38	0.07	*	-	-	2.96	0.03	*	87.59	1.13	e	0.10	0.01	e	3.04	0.28	*	0.11	0.01	e	3.26	0.32	e	0.21	0.02	e	6.11	0.59	e
ND - FD - SC-CO_2	3	6.73	0.17	*	-	-	5.89	0.13	*	87.43	0.47	*	0.21	0.03	*	3.15	0.32	*	0.21	0.02	*	3.13	0.26	*	0.42	0.04	*	6.28	0.59	e
ND - VD - SC-CO_2	3	8.98	0.18	*	-	-	7.83	0.07	*	87.18	0.90	*	0.28	0.04	*	3.16	0.40	*	0.30	0.02	*	3.30	0.10	*	0.57	0.05	*	6.36	0.41	*
**Pilot scale**																														
Non-disrupted	4	2.15	0.07	-	a	-	1.92	0.06	-	89.02	0.11	-	0.06	0.00	-	2.73	0.03	-	0.06	0.00	-	3.00	0.09	-	0.11	0.00	-	5.25	0.11	-
BM (1x)	3	2.01	0.04	*	a	-	1.78	0.04	*	88.60	0.42	e	0.06	0.01	e	2.87	0.23	e	0.06	0.00	*	2.96	0.18	e	0.11	0.01	e	5.57	0.35	e
BM (2x)	3	2.00	0.08	*	a	-	1.78	0.08	*	88.86	0.77	e	0.06	0.01	e	2.94	0.34	e	0.06	0.01	e	2.96	0.41	e	0.10	0.01	e	5.24	0.44	e
BM (3x)	3	1.88	0.34	e	a	-	1.66	0.32	e	88.54	1.17	e	0.06	0.00	e	3.41	0.75	e	0.05	0.01	*	2.53	0.08	*	0.10	0.01	e	5.52	0.54	e
BM - SD	3	1.85	0.11	e	b	-	1.64	0.09	e	88.83	1.10	e	0.05	0.01	e	2.77	0.24	e	0.05	0.01	e	2.57	0.36	e	0.11	0.02	e	5.84	0.58	e

[a)] n=Number of measurements

[b)] Ax/*H.p.*= Proportion of astaxanthin in relation to total *H. pluvialis* biomass (% w/w)

[c)] SD = Standard deviation

[d] Sig = Indicates significant differences (σ=0.05) between the sample and its predeseccor. All milled and high-pressure homogenized samples are compared to the undisrupted samples. * means significant difference and e indicates no significant difference. Samples highlighted in the same color were compared. Equal letter indicate no significant difference and unequal letters indicate significant differences.
[e] Prop. = Proportion of astaxanthin isomers in relation to total astaxanthin (% w/w)
[f] BM = Bead-milling
[g] SD = Spray-drying
[h] FD = Freeze-drying
[i] VD = Vacuum-dryning
[j] SC-CO2 = sSpercritical CO_2 extraction
[k] HPH = High-pressure homogenization
[l] ND = No disruption

Table S3: Estimated model coefficients, p-values and optimized parameters regarding maximal astaxanthin yield in spray-drying. Significant p-values (σ=0.05) are highlighted bold.

	**Linear model**		**Quadractic model**		**Quadractic model + interactions**	
	**coefficient**	**p-value**	**coefficient**	**p-value**	**coefficient**	**p-value**
a (Intercept)	1.8038		-0.1508		0.9095	
b_1 (Spray[a])	-0.001	**0.0011**	0.0046	0.2196	0.004	0.5303
b_2 (Feed[b])	0.0096	0.0848	-0.0123	0.6699	0.0797	0.749
b_3 (Temp[c])	-0.0017	0.1054	0.0065	0.6521	-0.0094	0.8376
b_{11} (Spray²)			0	0.1453	0	0.3004
b_{22} (Feed²)			0.0011	0.4536	0.0021	0.5842
b_{33} (Temp²)			0	0.6002	0	0.8778
b_{12} (Spray*Feed)					0	0.8265
b_{13} (Spray*Temp)					0	0.747
b_{23} (Feed*Temp)					-0.0005	0.6942
R^2	0.7151		0.8122		0.8244	
R^2 adjusted	0.6201		0.6244		0.2975	
**Optimized parameters**						
Feed	15		15		15	
Spray	400		400		400	
Temp	120		136.88		120	

[a]Spray = Spray gas flow (NL/h)
[b]Feed = Product feed rate (%)
[c]Temp = Inlet Temperature (°C)
